# Dancing with Nucleobases: Unveiling the Self-Assembly Properties of DNA and RNA Base-Containing Molecules for Gel Formation

**DOI:** 10.3390/gels10010016

**Published:** 2023-12-23

**Authors:** Pasqualina Liana Scognamiglio, Caterina Vicidomini, Giovanni N. Roviello

**Affiliations:** 1 Department of Sciences, University of Basilicata, Via dell’Ateneo Lucano 10, 85100 Potenza, Italy; 2 Institute of Biostructures and Bioimaging, Italian National Council for Research (IBB-CNR), Area di Ricerca Site and Headquarters, Via Pietro Castellino 111, 80131 Naples, Italy

**Keywords:** nucleobase, nucleopeptide, DNA, G-quadruplex, self-assembly, gel, hydrogel, triple helix RNA, G-quartet

## Abstract

Nucleobase-containing molecules are compounds essential in biology due to the fundamental role of nucleic acids and, in particular, G-quadruplex DNA and RNA in life. Moreover, some molecules different from nucleic acids isolated from different vegetal sources or microorganisms show nucleobase moieties in their structure. Nucleoamino acids and peptidyl nucleosides belong to this molecular class. Closely related to the above, nucleopeptides, also known as nucleobase-bearing peptides, are chimeric derivatives of synthetic origin and more rarely isolated from plants. Herein, the self-assembly properties of a vast number of structures, belonging to the nucleic acid and nucleoamino acid/nucleopeptide family, are explored in light of the recent scientific literature. Moreover, several technologically relevant properties, such as the hydrogelation ability of some of the nucleobase-containing derivatives, are reviewed in order to make way for future experimental investigations of newly devised nucleobase-driven hydrogels. Nucleobase-containing molecules, such as mononucleosides, DNA, RNA, quadruplex (G4)-forming oligonucleotides, and nucleopeptides are paramount in gel and hydrogel formation owing to their distinctive molecular attributes and ability to self-assemble in biomolecular nanosystems with the most diverse applications in different fields of biomedicine and nanotechnology. In fact, these molecules and their gels present numerous advantages, underscoring their significance and applicability in both material science and biomedicine. Their versatility, capability for molecular recognition, responsiveness to stimuli, biocompatibility, and biodegradability collectively contribute to their prominence in modern nanotechnology and biomedicine. In this review, we emphasize the critical role of nucleobase-containing molecules of different nature in pioneering novel materials with multifaceted applications, highlighting their potential in therapy, diagnostics, and new nanomaterials fabrication as required for addressing numerous current biomedical and nanotechnological challenges.

## 1. Introduction

Gels are three-dimensional networks of polymers and their swollen forms that cannot be dissolved in any solvents. In fact, they form structures acting as cross-linked networks that absorb solvents and swell to a limited extent without dissolving fully. Consequently, gels exist in a state that falls between a liquid and a solid. Gels are categorized by features such as the type of cross-linking forming their three-dimensional networks, their artificial or natural origin, and the types of solvents involved in the gelation process [1,2]. Gels can have various functions within living organisms, such as filtering, establishing molecular interactions between biopolymer chains and the enclosed solvent or solute [3]. Among the other types of gels, low-molecular-weight gels are a highly significant material category currently drawing considerable scientific attention. They emerge as small molecules that spontaneously assemble into linear structures interconnecting and intertwining to establish a network that can trap the solvent in its interior. The observed characteristics of gels rely on the method used for their obtainment, which leads to the creation of diverse materials possessing different properties starting from a single gelator. Moreover, using multiple gelators provides researchers the chance to realize materials with enriched information content and a broader spectrum of properties [4]. Apart from their undiscussed role in nanotechnology, gels are highly relevant also in food production, with various gelled food products being manufactured worldwide thanks to the ability of food gels to display useful viscoelastic properties [5,6,7]. Typically, the gelling agents that find application in food technology include proteins and polysaccharides. In food gels, polymeric structures are generally not held together by covalent interactions, except for the disulfide bonds found in certain protein-based gels. Instead, these structures are based on a combination of weak intermolecular interactions, such as Van der Waals forces, electrostatic forces, hydrogen bonds, and hydrophobic interactions. Polysaccharides, including the hydrocolloids used as thickeners in foods, possess noteworthy hydration properties in aqueous environments, but they often exhibit disordered structures [5]. The process of gelation depends on the nature of the gelling agent used as well as on the conditions under which gel formation occurs, including the pH, the presence of ions, the temperature, and the concentration of gelling agents. The characterization of gels is a process that involves various techniques, with rheological measurements being a routinely employed method. The development of mixed or multi-component gel systems is a noteworthy area of research, involving the simultaneous use of multiple gelling components in order to achieve specific structural and functional characteristics [5]. Polymeric gels are typically semisolid systems constituted by polymers, endowed with a three-dimensional network structure formed through either covalent or noncovalent bonding within a liquid medium, which accounts for the elastic properties of the resulting material. Among the key attributes of polymeric gels, it is worth mentioning their non-Newtonian (pseudoplastic) rheological behavior, as well as their properties of swelling, aging, syneresis, electrical oscillation, electrostatic potential distribution, electrical contraction, mechanoelectrical effects, and interaction with surfactants of opposite charge. In recent years, various types of polymeric gels (such as physical gels, cryogels, microgels, macrogels, nanogels, hydrogels, organogels, aerogels, emulgels, and xerogels) have been studied, developed, and utilized across diverse industrial strategies [8]. While the terms “gel” and “hydrogel” both refer to materials with gel-like characteristics, they differ in their composition and properties, particularly owing to their interaction with water. Gels can be composed of diverse substances, including polymers or small molecules, whose cross-linking forms a network immobilizing liquid within its structure. They exhibit properties of both liquids and solids, having an overall semi-solid consistency together with the ability to hold a significant amount of liquid within their structure without dissolving. Gels can be formed by chemical or physical cross-linking of their components, which leads to different behaviors and characteristics. As for hydrogels, this is a specific class of gels with a strong affinity for water or, in other terms, high hydrophilicity, as indicated by the word “hydro” in hydrogel, which refers to the high water content effectively found in these substances. In fact, hydrogels are mainly composed of a network of hydrophilic polymer chains and water. The usually cross-linked networks of polymers can absorb and retain a significant amount of water without losing their structural integrity. Hydrogels find application in numerous fields including drug delivery, tissue engineering, wound dressings, contact lenses, and personal care products. Moreover, they can be engineered to acquire specific characteristics linked to their high water content, their ability to respond to stimuli like the pH and temperature, but also their biocompatibility. Hydrogels may retain a specific three-dimensional framework and were initially investigated as biomaterials for human body applications [9,10,11]. Techniques routinely used in biomaterial synthesis involving reactive polymer precursor crosslinking, polymer–polymer reactions, and crosslinking copolymerization were successfully applied to create hydrogels. However, it was not easy achieving precise structural control due to side reactions resulting in network complexities like unreacted groups and intertwinements. Additionally, conventional hydrogels suffered from limits such as suboptimal mechanical characteristics and weak responsiveness to external stimuli. This called for a significant innovation in hydrogel formulation aimed at reinvigorating the research on these materials with contemporary attempts to create hydrogels endowed with enhanced mechanical strength as well as convenient superporous characteristics and graft structures able to respond promptly to external stimuli. In this regard, interesting examples employing self-assembling hydrogels derived from hybrid graft copolymers containing protein domains and others realized from genetically engineered triblock copolymers were reported in the scientific literature, shaping a promising future for smart hydrogel biomaterials [12]. In this review, we will describe the self-assembly properties of different molecules all containing nucleobases, with a particular focus on the gel formation properties of some of these compounds. In particular, we will present some useful features of nucleobase-bearing amino acids (indicated as nucleoamino acids), nucleoamino-containing peptides (nucleopeptides), as well as natural nucleosides (such as guanosine) and nucleic acids (DNA and RNA). In the case of DNA and RNA, particular structures involving the double helix, triple helix, and quadruple helix (G-quadruplex) topologies also find mention as they are implied in self-assembly processes leading in some cases to gel formation, as will be described below. More in detail, several nucleobase-containing chemical structures have been identified for their capability to form gels or hydrogels due to their unique nucleobase-driven molecular binding and self-assembly properties. Some of these include peptide nucleic acids (PNAs) [13], which are synthetic nucleic acid analogs with a peptide backbone endowed with numerous biomedical properties [14,15]. Certain structurally modified PNAs have been reported to form hydrogels, enabling the realization of materials with diverse functionalities [16,17]. Oligonucleotides with modified nucleobases or chemical modifications of the sugar-phosphate backbone have also been reported to self-assemble into gels or hydrogels [18,19,20]. Overall, modified oligonucleotides enable the creation of self-assembling materials with tailored properties and enhanced stability. As for the importance of the nucleobase’s nature in the gel formation process, certain guanine derivatives and, particularly, guanosine-based small molecules and polymers have been explored for their ability to form guanosine-containing supramolecular structures and gels through hydrogen bonding and π–π stacking interactions [21,22,23,24]. Apart from the oligomeric structures of DNA and their analogs, the monomeric nucleosides or nucleotides modified with non-natural bases, including fluorinated nucleosides or other base analogs, or sugar modifications have also been explored for their gel-forming properties [25,26,27,28]. The design and construction of nucleobase-containing structures capable of forming gels or hydrogels involve the promotion of interactions such as hydrogen bonding, π–π stacking, or other supramolecular interactions by chemical modification of the nucleobase-containing structures. These materials have gained considerable attention due to their potential applications in various fields, including biotechnology, drug delivery, and tissue engineering. Thus, in this review, we focus on nucleobase-containing compounds and their inherent self-assembly characteristics, placing special emphasis on their ability to form gels. This discussion covers the main characteristics of nucleopeptide gels and diverse types of polymeric gels derived from nucleic acids or other nucleobase-containing structures such as mononucleosides, encompassing gel’s physical and biomedically relevant properties, as described in the subsequent sections.

## 2. Nucleobase-Bearing Amino Acid Systems and Self-Assembly

Nucleobase-containing amino acids, also known as nucleoamino acids [29], consist of amino acid residues linked to DNA or RNA bases through diverse connecting moieties referred to as linkers. These hybrid derivatives, featuring heteroaromatic rings fused with amino acid-based structures, are sourced from nature or synthesized by means of chemical procedures in a laboratory. Notably, among the several natural nucleoamino acids worth mentioning is willardiine, which can be described as a uracil-bearing alanine, as well as amino nucleosides like cystocin and puromycin, recognized for their antimicrobial properties. These chimeric compounds can be seen as a bridge between the families of nucleobases and amino acids playing a significant biological role and, from a synthetic perspective, serve as fundamental components for building up peptides with intriguing DNA-binding capabilities. In the class of natural nucleoamino acids, willardiine and its analogs stimulate AMPA or kainate receptors [30], while discadenine [31], derived from *Dictyostelium discoideum*, impedes self-spore germination and acts as a plant cytokinin. Also, lathyrine, a non-proteinogenic amino acid found in several *Lathyrus* species and identified as a potential food allergen, belongs to the nucleoamino acid family [32]. Additionally, synthetic nucleoamino acid monomers serve as building blocks for fabricating the nucleobase-incorporating peptides often referred to as nucleopeptides [33,34,35,36,37,38,39,40]. Further examples of natural nucleobase–amino acid conjugates are evident in antimicrobial peptidyl nucleosides [41] and in the aminoacyl nucleoside *N*6-threonylcarbamoyl adenosine [42], present in transfer RNA and involved in the protein synthesis process. Both naturally occurring and artificially synthesized nucleobase-containing amino acids are obtainable through chemical synthesis. Similar to their nucleoamino acid constituents, nucleobase-decorated peptides also constitute a promising class of molecules with significant scientific value proving beneficial in biotechnology and medicine, as they merge a peptide-like structure with DNA or RNA nucleobases connected through diverse linkers. Interestingly, among these chimeric compounds, only a limited number were shown to occur naturally, such as willardiine-containing short nucleopeptides and peptidyl nucleosides, also referred to as nucleoside peptides, renowned for their antimicrobial and anti-tumoral properties [43]. Conversely, several research groups have prepared and investigated artificial nucleobase-containing peptides, or pseudopeptides, paying particular attention to the study of their binding characteristics. Notably, certain nucleobase-containing peptides demonstrated a capability to bind complementary DNA and RNA, with intriguing prospects for biomedical applications in antigen and antisense strategies. Gel polymer systems rely on the assembly of one or multiple types of monomeric subunits held together through non-covalent interactions. Within the various range of molecules capable of constructing supramolecular networks, particular attention is directed towards nucleobase-containing molecules. These include not only nucleic acids, which are widely used in nanomedicine, but also the above-mentioned nucleobase-containing peptides and systems composed of individual monomeric units carrying single nucleobases. Supramolecular materials based on nucleopeptides have also attracted significant interest, particularly due to their versatility in forming various nanostructures.

**Scheme 1:**
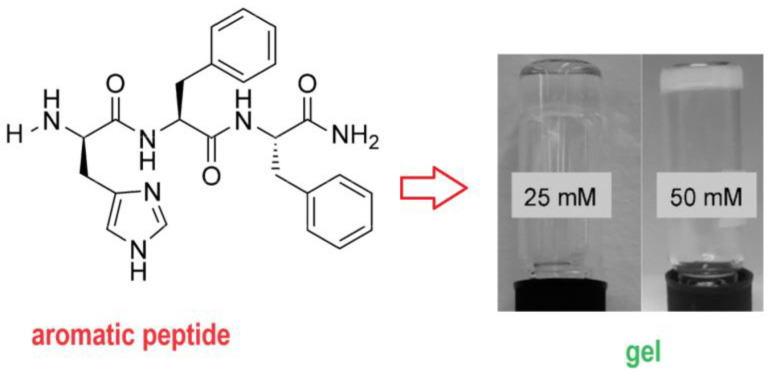
Schematic representation of gel formation starting from an aromatic peptide gelator. The photographs refer to the gel obtained from the tripeptide l-His–d-Phe–d-Phe in a phosphate buffer at 25 and 50 mM. The pictures were adapted from https://www.mdpi.com/1420-3049/26/1/173 (accessed on 15 December 2023).

On the other hand, peptides, especially those based on aromatic moieties such as the tripeptide l-His–d-Phe–d-Phe, and amino acid derivatives, both extensively utilized structures, possess inherent properties suitable for nanotechnological, biological, and medical applications, which are, at least in part, attributed to their remarkable self-assembly properties, leading to useful applications including in the formation of hydrogels (Scheme 1) [44].

**Figure 1:**
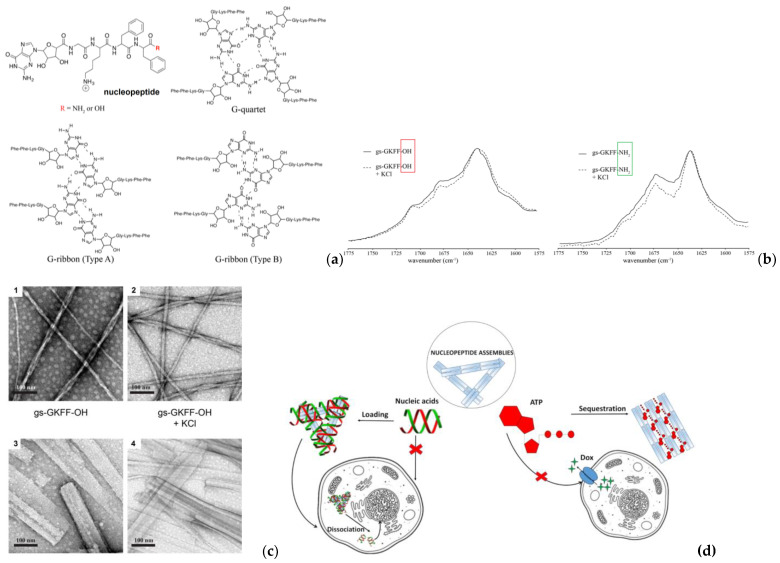
Nucleopeptides for self-assembling materials. (**a**) Nucleopeptide structure and potential secondary structures based on guanosine. The nucleopeptide was created through the modification of the short peptide Gly-Lys-Phe-Phe by incorporating guanosine mononucleoside. Two variants of nucleopeptides, one with an amide and the other with a carboxylic acid C-terminal, were examined. The proposed structures for assembly using guanosine involve either a G-quartet or G-ribbon architecture. (**b**) A FTIR analysis was conducted for assemblies of the nucleopeptides gs-GKFF-OH (**left**) and gs-GKFF-NH_2_ (**right**) after one week of assembly, in the absence of KCl (solid line) and in the presence of one equivalent KCl (dashed line). (**c**) TEM images obtained after one week of assembly for gs-GKFF-OH without KCl (**1**) and with 10 equivalents of KCl (**2**). Similarly, the TEM images were taken for gs-GKFF-NH_2_ after one week of assembly without KCl (**3**) and with 10 equivalents of KCl. (**4**). (**d**) Examples of applications of nucleopeptide assemblies in biomedical strategies of gene delivery (**left**) and ATP sequestration from cancer cells (**right**). For more details please visit the links below. The pictures in (**a**–**c**) were adapted from https://www.mdpi.com/1420-3049/25/23/5493 (accessed on 28 November 2023), while those shown in (**d**) were from https://www.mdpi.com/1420-3049/26/12/3558#B90-molecules-26-03558 (accessed on 14 December 2023).

For gel formation, the mono-component approach involving a single molecule is widely used due to its simplicity, but this strategy is not exempt from drawbacks in terms of the resulting properties of the obtained gel system. To overcome these limitations, the multi-component approach emerged as a promising strategy employable when realizing gels starting from low-molecular-weight molecules that interact with each other to generate novel assemblies and properties unattainable with a single gelator. In this context, a series of nucleopeptides, integrating both peptide and DNA-nucleobase components covalently linked with each other, was investigated for gel formation. Both hydrogels and soluble supramolecular networks hold promise for delivering genes and drugs to cells. These nucleopeptide-based systems were found to be able to originate gels through a cooperative effect facilitated by the presence of nucleobases [45]. Famously, phenylalanine (Phe) is an aromatic amino acid, present in proteins, that forms a dipeptide (Phe–Phe) capable of self-assembling into innovative nanomaterials [46,47]. In particular, when in a solution the dipeptide self-assembles into fibers, reliant on π–π interactions, in turn responsible for generating tubular structures, serving as templates for the formation of nanotubes. In this context, investigating the chemical modification of Phe–Phe and, in particular, the substitution of one phenyl ring in the dipeptide with different aromatic or heteroaromatic groups is an intriguing possibility, potentially influencing both aggregation and biomolecular interactions with natural targets. In this context, an experimental study aimed to synthesize and investigate the properties of PheT, an aromatic nucleobase amino acid derived from Phe and thymine nucleobase, mimicking Phe–Phe but with one aryl group substituted by the T nucleobase [48]. In the design of PheT, the Phe component contributed aromatic and hydrophobic interactions, crucial for the self-assembly properties of the construct, while the nucleobase segment potentially enabled interactions with biomolecules. The arrangement of the two aromatic rings disfavors direct stacking, allowing both phenyl and thymine units to interact with residues from other molecules (e.g., nucleic acids, proteins, or other PheT units). This study demonstrated the effective occurrence of such interactions through circular dichroism (CD) and UV spectroscopies, along with binding assays involving DNA, RNA, and protein models [48]. Furthermore, another work reported on the design, synthesis, and characterization of a nucleoamino acid derivative named TrpT, based on L-tryptophanamide functionalized with a thymine nucleobase [49]. We can see TrpT as another analog of the Phe–Phe in analogy to PheT with tryptophan and thymine being in place of the two Phe residues. The TrpT molecule demonstrated a clear propensity to self-assemble into supramolecular networks in aqueous solutions, as verified through dynamic light scattering (DLS), CD, fluorescence, and UV spectroscopy. The nucleoamino acid underwent self-assembly into nanoaggregates, driven mainly by thymine–thymine π−π stacking, likely exposing the tryptophan moieties of the outer layers to the aqueous environment and forming hydrophobic pockets which include other tryptophan units within the particle interior. The ability of TrpT to create nanostructures in a solution was validated through different in silico and experimental analyses, revealing predominantly spherical species with good stability. Notably, a confocal fluorescence microscopy analysis demonstrated that the supramolecular TrpT network had the capacity to accommodate and subsequently release hydrophobic drugs gradually, suggesting its potential as a drug delivery system. These nanoaggregates exhibited stability for up to 5 h at a concentration of 140 µM, displaying a mean hydrodynamic diameter of 330 nm and uniform size distribution, as observed via scanning electron microscopy (SEM). Moreover, the ability of TrpT nanoaggregates to bind to the natural anti-cancer curcumin, serving as a model drug, was assessed in the same study, and the natural drug release properties of the non-covalent polymer were eventually demonstrated using confocal microscopy [50]. Molecular docking studies indicated the accommodation of curcumin within the interior of the TrpT nanoaggregates in which the curcumin was bound through hydrophobic interactions. Additionally, the stability of the TrpT nanoassemblies in human serum and their minimal toxic effects on human model cells were revealed in the same study. Quite surprisingly, despite its thymine-based structure, TrpT did not exhibit any appreciable binding towards adenine-rich nucleic acids, suggesting a preference for self-assembly over A-T base pairings. Moreover, TrpT did interact with a serum protein, bovine serum albumin (BSA), known for enhancing the transport and bioavailability of its biomolecular cargos in the bloodstream. Overall, these findings suggested the potential utility of TrpT nanosystems in the development of new drug delivery systems [49]. Apart from these studies on non-covalent polymers based on nucleobase–amino acids conjugates, more applicative works were conducted on hydrogelators obtained through the fusion of nucleobases and short peptides, which showed that nucleopeptides, thanks to their self-assembly in aqueous environments, are able to form supramolecular hydrogels upon stimulation by enzymatic action or pH variations [51]. Notably, nucleopeptides may offer easy and broadly applicable methods for producing biocompatible structures, and the simplicity of incorporating various bioactive peptides or molecular recognition elements together with nucleobases opens new avenues for exploring innovative applications of nucleopeptides as functional biomaterials [51]. Short peptide sequences sourced from the interface of a known heterodimeric protein were combined with nucleobases, thus forming nucleopeptides which were found to predominantly self-assemble via hydrogen bonds, leading to the creation of nanofibers, ultimately leading to supramolecular hydrogels obtained by merely mixing two nucleopeptide samples in water [52]. Additionally, apart from demonstrating its biocompatibility with mammalian cells, the nucleopeptide heterodimer exhibited a noteworthy resistance against proteinase K proteolysis, which is a favorable characteristic in view of the biomedical applications of biomaterials obtained via the supramolecular hydrogelation of heterodimeric nucleopeptides [52]. Self-assembled nucleopeptide hydrogels were shown to form nanofibril architectures by means of noncovalent interactions, including Watson–Crick interactions and π–π stacking, the former being facilitated by the presence of complementary nucleobases in the structures. These hydrogels are envisaged to offer specific advantages for biomedical applications, combining the easily modulable DNA-interacting mode [53] to the well-established advantages of peptide biomaterials, such as their customizable design, biocompatibility, and extracellular matrix-like structure. Drawing inspiration from their nucleobase-stacking structure, the capability of nucleopeptides to provide sustained delivery of the DNA-intercalating chemotherapy drug doxorubicin when locally administered to a solid tumor was assessed using an in vivo tumor-bearing mouse model [54]. This demonstrated that an adenine-bearing triphenylalanine (Ade–FFF) nucleopeptide was able to form hydrogels with a high loading capacity for doxorubicin at a 1 mM concentration, exhibiting the drug’s continuous release under in vitro degradation conditions [54]. Doxorubicin-loaded Ade–FFF hydrogels decreased the tumor growth levels and enhanced apoptosis-mediated cell death within the tumor, as indicated by caspase-3 expression. Biodistribution and pharmacokinetic analyses further supported the observation that delivering the drug through the nucleopeptide hydrogel increased the levels of sustained release specifically at the local tumor site in the animal model. This investigation highlights the potential of self-assembled nucleopeptides for various biomedical applications by taking advantage of their unique dual DNA-like and peptide structural features [54]. Over the past few decades, there has been a considerable focus on peptide-based hydrogels as versatile supramolecular materials, offering novel possibilities for various biomedical applications. To gain deeper insights into their self-assembly properties and enhance their characteristics, we have at our disposal several strategies including modifying the amino acid chains by incorporating halogenated amino acids, pseudopeptide bonds, or other chemical moieties into the peptide backbone. In this respect, integrating DNA-nucleobases into peptide scaffolds leads to the development of new examples of nucleopeptides. In previous studies, some of these hybrid molecules led to the formation of nucleopeptide hydrogels whose physicochemical and mechanical properties strongly relied on the specific nucleobase introduced in the structure (whether cytosine, thymine, guanine, or adenine) [55]. The hydrogel properties resulting from this process can be improved and precisely adjusted, leading to an enhanced resistance to external stress, significant increases in gel stiffness, and the emergence of distinctive thermo-reversible and red-edge excitation shift properties. The precise contributions of each nucleopeptide component in the self-assembly processes can be proven using an array of analytical techniques such as NMR relaxometry, rheology, TEM, fluorescence, CD, FTIR, NMR chemical shift index, and thioflavin T assays, ultimately demonstrating that nucleopeptide hydrogels offer novel opportunities for tailoring hydrogel properties according to specific requirements [55]. Nucleopeptides can be seen as innovative chimeric compounds resulting from the fusion of nucleobases and peptides [56], typically self-assembling into nanofibers driven primarily by hydrogen bonds and other weak forces. Their unique characteristic involves the potential ability to bind complementary nucleic acids due to the presence of nucleobases in the nucleopeptide structure. Thus, nucleopeptides are the subject of research as building blocks capable of self-assembling in water and as artificial oligonucleotides able to target single-stranded DNAs or RNAs. Towards DNA, specific nucleopeptide structures have been demonstrated to be able to bind with good affinity to single-stranded DNAs, which reciprocally influenced each other’s self-assembly abilities [57]. Not less importantly, certain nucleopeptides have been found able to interact with plasmid DNAs and facilitate the delivery of hairpin DNA to cells [57]. In other works, other nucleopeptide analogs resembling TpT dinucleoside monophosphate generated supramolecular networks interconnected via noncovalent interactions. These non-covalent polymers exhibit the capacity to accommodate organic molecules, demonstrating potential applications in drug and gene delivery [58]. Considering the significance of these molecular systems in the field of biomedicine, nucleopeptide analogs of mononucleosides were also investigated in studies aimed to ascertain their capacity to construct supramolecular networks using these short nucleopeptides, intending to develop innovative drug delivery strategies. More in detail, thymine, adenine, cytosine, and guanine nucleoside analogues were synthesized and subjected to investigation with respect to their supramolecular assembly properties. Chemically, the four nucleopeptides originated from a diserine (Ser–Ser) peptide conjugated to a DNA nucleobase present at its *N*-terminus. These structures were investigated by light scattering and CD studies evaluating their interactions with natural nucleic acids and assessing the formation of supramolecular networks based on nucleobase recognition. The results revealed the formation of molecular networks held via weak interactions (such as hydrophobic, hydrogen-bonding, and aromatic interactions), with structural changes being influenced by temperature fluctuations. The same peptidyl nucleoside analogues demonstrated good biodegradability properties and led to the formation of supramolecular complexes involving multiple nucleopeptide units alongside with nucleic acid molecules [59]. Due to their inherent properties and flexibility in terms of structure, low-molecular-weight gelators, particularly peptide-based hydrogelators, have great importance, as the resulting supramolecular hydrogels are readily obtained from specific self-assembly of the peptide constituents, modulable through tailored chemical modifications applied to the peptide structure. Among these chemical modifications, the introduction of nucleobases, in turn constituting an additional family of biomolecules renowned for their self-assembling properties, has emerged as an attractive strategy employed to design supramolecular hydrogels based on low-molecular-weight nucleopeptides [60]. Thanks to their dual nucleic acid–peptide properties, the nucleopeptides were often found able to co-assembly, with complementary nucleobase segments interacting through π-stacking interactions and hydrogen bonding, which resulted in synergistic effects, as proven by nucleopeptide structures in which a tetrapeptide moiety was conjugated with a two-bases-long nucleopeptide. These effects enhanced the mechanical properties of the resulting hydrogels by over 250%, with stiffness levels over 700 kPa, and elevated their self-assembling abilities by approximately 280%. The structure-to-property correlations for the above-mentioned systems were investigated through a comprehensive analysis based on multiple techniques, such as fluorescence, rheology, transmission electron microscopy (TEM), cryo-SEM, FTIR, NMR, CD, and high-resolution magic angle spinning. This in-depth analysis indicated the influence of nucleobases on the supramolecular assembly process, on the consequent formation of nanostructures, and on the three-dimensional structure of the hydrogel scaffold as well as on the resulting physical and mechanical characteristics of these synergistic nucleopeptide assemblies. Different from most hydrogels derived from single gelators through mono-component strategies, often associated with several limitations, the nucleopeptide co-assembling system demonstrated a high efficiency, yielding high storage moduli (about 720 kPa), and noteworthy synergistic physical and mechanical nanomaterial properties. Specifically, the nucleopeptides presented cooperative effects, especially when combining two complementary sequences, TG–FEFK and AC–FEFK, where TG and AC are the dibasic nucleopeptide moieties linked to the FEFK tetrapeptide. These nucleopeptide sequences resulted clearly complementary thanks to the complementary nucleobase-bearing segments. This nucleopeptide combination significantly improved hydrogel stiffness and resistance to external stress, respectively, with their resulting properties greatly overcoming those associated with the individual nucleopeptides or the systems obtained mixing samples of nucleopeptides with mismatched nucleobases. The observed synergy stems from specific interactions occurring between the peptide segments (evidenced by π-stacking interactions and β-sheet formation) and between the base-containing moieties via hydrogen bonding through complementary nucleobase pairing and additional π-stacking. The resulting supramolecular interactions led to a fibrillary network capable of self-organizing into a porous hydrogel scaffold endowed with uniform alveoli, efficaciously entrapping water molecules in their interior, as demonstrated through TEM and cryo-SEM. Taken together the above findings suggested the significant potential of nucleopeptides as a highly efficacious element in forming low-molecular-weight hydrogels. Remarkably, owing to their unique dual nucleic acid–peptide nature, nucleopeptides offer new opportunities for designing multi-component hydrogels with remarkable synergistic effects providing significantly improved mechanical and physicochemical properties that would otherwise be challenging to achieve through a traditional mono-component approach [38]. Numerous are the applications that self-assemblies and gels of nucleopeptides can provide [61]. The capability of nucleopeptides and PNA to create well-organized architectures was proven and the obtained supramolecular tools, including nanovesicles, nanotubes, nanospheres, nanofibers, or micelles (including cylindrical, spherical, or worm-like structures), exhibited applications in biomedicine, nanotechnology, or materials science due to their favorable properties of biocompatibility and biodegradability. For instance, by taking advantage of the non-covalent interactions between nucleic acids and nucleopeptides, different researchers developed nucleopeptide-based supramolecular assemblies designed for gene delivery therapy [62] or for the selective sequestration of ATP in cancer cells (as illustrated in Figure 1). Displaying minimal cytotoxicity by themselves, the nucleopeptide systems were found to significantly increase the cytotoxicity of doxorubicin used as anti-cancer drug against human uterine sarcoma cells in a dose-dependent manner [63]. This approach enhances the effectiveness of anti-cancer drugs such as doxorubicin and offers unique advantages, including reversible interactions between assemblies and nucleic acids, minimal immunogenicity, and biocompatibility.

Other multi-component self-assembling hydrogels disclosed new scenarios in creating materials with various properties that would be challenging to achieve using individual components alone. Consequently, these multi-component-derived hydrogels are envisaged to serve for wide-ranging applications in biomedicine, and the numbers of examples with such systems will probably continue to grow. Multi-component self-assembly strategies were applied to develop a biomimetic, low-molecular-weight guanosine quartet-based hydrogel under physiological conditions. The introduction in the nucleopeptide structure of the mononucleoside guanosine and the use of 4-formylphenylboronic acid and a cytosine-functionalized nucleopeptide are paramount to creating dynamic imino–boronate ester-mediated G-quartet-based hydrogels. The effective formation of a G-quartet structure, a crucial factor leading to nanofibrillar hydrogels, was demonstrated through CD, powder X-ray diffraction, and thioflavin T fluorescence assay. The multi-component self-assembled G-quartet-based hydrogel exhibited remarkable antibacterial activity against a number of bacterial species. The in vitro cytocompatibility of the hydrogel was proven on HEK 293T and MCF-7 cell lines, revealing the biocompatibility of such G-quartet-based hydrogels which, overall, were demonstrated to be injectable, biocompatible, and intrinsically antibacterial materials holding promise for preventing localized microbial infections [64]. In another experimental work, guanosine-containing self-assembling nucleopeptides (Figure 1) were able to give rise to nanofibers and nanosheets. By taking advantage of spectroscopy and microscopy techniques, the assembly into β-sheet structures of such G-based nucleopeptides was proven, which primarily occurred thanks to the peptide moiety present in the nucleopeptide structure, whereas the hydrogen-bonded guanosine elements contributed to the formation of additional secondary structures, cooperatively embedded within the peptide structure (Figure 1). Notably, the observed supramolecular nucleopeptide morphologies were not dependent on the metal cations, whose responsiveness is typically observed in guanine-based nanomaterials, but rather on the influence of the peptide moiety present at the nucleopeptide’s *C*-terminus. Overall, the presented research underscores the structural diversity shown by self-assembling nucleopeptides and suggests new progresses on applications associated with these supramolecular G-containing nucleopeptides [40]. With all the above being said, we can conclude that hydrogels formed by nucleopeptides able to self-assemble offer substantial advantages in different biomedically relevant fields owing to their biocompatibility and broad spectrum of molecular possibilities. The short peptide moieties present in nucleopeptides, in particular, offer remarkable advantages, including their easy synthesis and the favorable self-assembly properties conferred to the resulting nucleopeptide. While the biomedical applications of classical peptides are currently limited due to challenges such as the potential toxicity resulting from the chemical modifications of natural peptides required for their self-assembly as well as from the experimental conditions required for gelation, one possibility at our disposal to mitigate their cytotoxicity involves the conjugation of peptides to nucleobases, which leads to nucleopeptide structures. Nucleopeptide hydrogel formation can be achieved under specific conditions and can be easily modulated using salts and biological buffers. In this regard, the self-assembly of nucleopeptides relies on the experimental conditions adopted and can be regulated by their formulation and pKa. In solutions adjusted to physiological values of osmolarity and pH which are compatible with cell culture, hydrogel formation is often favored. In silico and analytical methods can be employed to explore the effects of salts and pH conditions on nucleopeptides at the molecular and structural levels. Thanks to the specific mechanisms governing the self-assembly of nucleopeptides, one can modulate nucleopeptides’ mechanical properties through the addition of divalent cations, which leads to an increased hydrogel storage modulus. The stability of nucleopeptide hydrogel constructs offers a potential for long-term cell culture, with the survival and proliferation of fibroblasts having been shown on the surfaces of these hydrogels. The nucleopeptide hydrogelation methodology mediated using biological buffers makes way to tissue-engineering applications involving nucleopeptides [65]. In this respect, self-assembling nucleopeptides offer a methodical strategy for building hydrogels resembling the extracellular matrix in both function and structure, as shown with certain nucleo-tripeptides capable of forming hydrogels under physiological conditions. Combining experimental and in silico methods, their self-assembled structures were examined using CD spectroscopy, TEM, and rheometry methods employed to validate and complement the computational results obtained with molecular dynamics simulations. The nucleo-tripeptides were shown to form hydrogels based on nanofibers held via interactions including π–π stacking and Watson–Crick complementary base pairing. The conditions for self-assembly were modulated thanks to the hydrophobic and amphiphilic moieties present in the structures of the nucleo-tripeptides, with new possibilities offered for deliberate control using a rational molecular design. Overall, structures arising from nucleobase-containing peptides and their combinations are capable of forming hydrogels under physiological conditions, highlighting them as promising candidates for innovative biomedical applications [66].

## 3. Nucleic Acid-Based Gels

**Figure 2:**
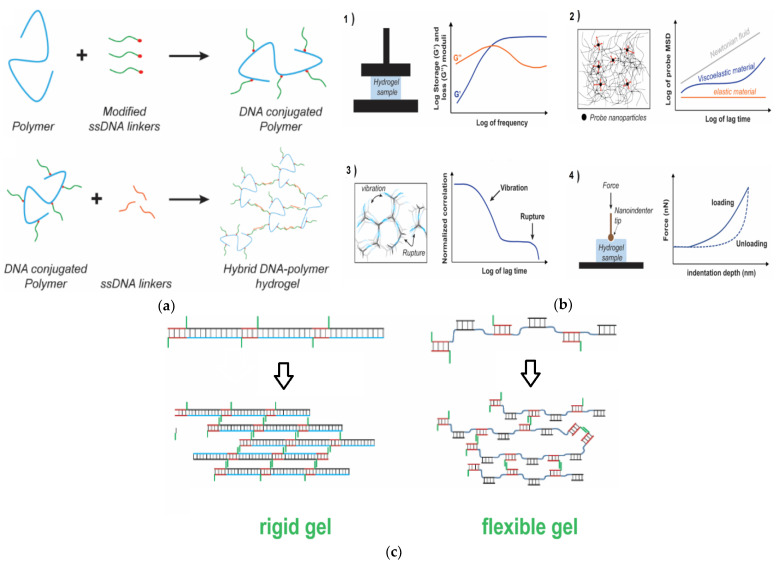
(**a**) Hybrid DNA–polymer hydrogel formation. Polymer chain conjugation with modified single-stranded DNA linkers to form single-stranded-DNA-conjugated polymer. Single-stranded-DNA-conjugated polymers crosslinked by complementary single-stranded DNA linkers to form hybrid DNA–polymer hydrogel. (**b**) Common characterization methods for DNA hydrogel. (**1**) Bulk rheology and dynamic moduli. (**2**) Graphical depiction of microrheology showing constrained probe particle diffusion within the hydrogel network. The probe mean squared displacement (MSD) is used to determine the material’s behavior. (**3**) Network vibrations and interparticle hybridization rupture measured using dynamic light scattering (DLS) with fast and slow decay times being observed and corresponding to the timescale for each event, respectively. (**4**) Probe-based force-controlled nanoindentation of DNA-based hydrogels to determine the material behaviors. (**c**) Structural design of DNA hydrogels involving the arrangement of hybridized linear segments of double-stranded DNAs leading to rigid or flexible hydrogels. For more details please visit the links below. Pictures in (**a**–**c**) were adapted from https://www.mdpi.com/2076-3417/11/4/1885 (accessed on 28 November 2023).

DNA hydrogels are unique biomaterials resulting from the self-assembly of DNA into three-dimensional frameworks. Certain types of DNAs, endowed with “sticky-end” sequences, are capable of forming DNA hydrogels thanks to molecular interactions such as base stacking and complementary base pairing [67]. In other terms, DNA hydrogels often originate from short DNA sequences that contain complementary base sequences at their ends. In fact, when these oligonucleotide sequences come into contact, they hybridize, forming double-stranded DNA structures, a process which also causes the assembly of three-dimensional DNA supramolecular networks. Different DNA motifs have been explored for their ability to form hydrogels and were variously engineered in order to display suitable complementary regions provoking their self-assembly and formation of stable structures under specific conditions of pH, ionic strength, and temperature. The ability of DNA to form hydrogels can have numerous applications in various fields, ranging from tissue engineering and biosensing to drug delivery, also thanks to their modulability and biocompatibility. At a molecular level, specific DNA sequences can be designed to create DNA hydrogels, and, thus, new efforts are being continuously devoted to exploring new possibilities for creating versatile and functional DNA gel-based materials. Cationic surfactants based on amino acids were used as innovative biocompatible tools for controlled DNA encapsulation and subsequent release. DNA gel particles were formed through the combination of DNA, specifically single-stranded or double-stranded DNA, with two different single-chain amino acid-based surfactants: arginine-*N*-lauroyl amide dihydrochloride, and *N*α-lauroyl-arginine-methyl ester hydrochloride [68]. DNA entrapment, swelling/deswelling behavior, and the release kinetics of DNA were assessed considering factors such as the number of charges within the nucleic acid’s secondary structure and the surfactant’s polar head. A more pronounced interaction was found between the first surfactant and DNA compared to the second one, which can be mainly explained on the basis of the double charge in the former surfactant’s headgroup compared to the singly charged headgroup of the latter species. Moreover, the stronger interaction observed with amphiphiles for single-stranded DNA, as opposed to double helical DNA, seemed to indicate that hydrophobic interactions have an effect on DNA dynamics. Using small-angle X-ray scattering it was possible to demonstrate a hexagonal arrangement in the microstructure of the complexes obtained from the particles. Remarkably, a stronger interaction between the surfactant and DNA was correlated with a shorter lattice parameter, which resulted in a slower rate for the release of DNA. Agarose gel electrophoresis measurements demonstrated the binding and neutralization of DNA within the gel particles [68]. DNA-based supramolecular hydrogels can also be defined as hydrogels that are formed through DNA hybridization. These materials have attracted significant interest for their exceptional biocompatibility, molecular permeability, thixotropic behavior (i.e., their viscosity decreases under mechanical stress, passing from a more solid to a more fluid state), self-repair capability, and degradability. These properties render them particularly useful in various fields, such as tissue engineering, 3D printing, and cell culture. Notably, the programmable nature and responsiveness of DNA enable the DNA hydrogels to respond to suitable external stimuli, expanding the potential applications of such gels to include shape-memory materials and detection devices [69]. As for the forces responsible for DNA hydrogel formation, these biomaterials self-assemble mainly by means of Watson–Crick base pairing, which leads to complex three-dimensional frameworks based on nanostructured DNA building blocks (Figure 2a). Due to the unique biochemical, mechanical, and biocompatibility characteristics of DNA, DNA hydrogels characterized using different methods (Figure 2b) offer controlled composition and mechanical properties, presenting opportunities in various biomedical applications and leading the way to the creation of new-generation multifunctional biomaterials. 

Mechanical properties of DNA gels can be modulated by creating pH-responsive DNA-based hydrogel microcapsules. Additionally, the introduction of thermal-responsive units into the network allowed the fabrication of thermally responsive DNA hydrogels. Reversible mechanical strength was also achieved in a DNA hydrogel by incorporating poly (propylene oxide) as a thermal-responsive unit into DNA networks. By doing so, the mechanical strength of the DNA hydrogel was found to change reversibly between 218.2 and 503.5 Pa when the temperature varied between 37 and 4 °C [70]. While integrating DNA with polymers may enhance the modulability of hydrogels’ properties and functions, the use of synthetic polymeric materials may affect their biocompatibility. On the other hand, pure DNA hydrogels, without synthetic or other natural polymers, offer programmable structural characteristics that could further benefit their applications. For instance, the integration of particular DNA topologies such as i-motif sequences into a DNA hydrogel network leads to a reversible modulation of the hydrogel’s mechanical strength ranging from 250 to 1000 Pa [70]. Similarly, by incorporating an ATP aptamer into the linker DNA, the mechanical strength of DNA hydrogels increased from 204 to 570 Pa. Moreover, the combination of dynamic and permanent DNA crosslinking networks resulted in the fabrication of super-soft and super-elastic magnetic DNA hydrogels [70]. Owing to porosity of DNA hydrogels, carbon nanotubes (CNTs) play a pivotal role, tuning the porous structure of a hydrogel but also enhancing the material’s adsorption properties. Incorporating CNTs into the DNA/CNT hybrid gel and organizing it into a porous framework led to a substantial increase in the swelling rate and capacity of the resulting material [71]. Recent studies have shown that DNA hydrogels are functional materials responsive to various stimuli, including light, pH, biomolecules, and temperature. Several investigations have focused on features of DNA hydrogels such as functionality, chemical crosslinking, and linker flexibility, which significantly influences the hydrogels’ macroscale mechanical properties. Pure DNA hydrogels and their hybrid combinations find application as matrices for cell culture, which unlocks their potential as highly adaptable and tunable biomaterials endowed with multifaceted functionalities [72]. DNA hydrogel design should take into account characteristics such as their responsiveness to stimuli and their mechanical properties in order to obtain gels utilizable as functional biomaterials. In particular, to achieve tunable macroscale mechanical properties great attention is currently paid to DNA hydrogel nanoscale design, even though establishing standardized design principles for obtaining such nucleic acid-based hydrogels as biomaterials endowed with specific mechanical attributes remains challenging due to the vast design possibilities offered by DNA supramolecular biosystems. Among the most critical factors for the design of DNA hydrogels with specific properties, the steric availability of crosslinking points, their structural rigidity, and their melting temperature (T_m_) came to prominence. Other factors to be considered in DNA hydrogel design include the total DNA content for the high costs associated with large-scale DNA synthesis. Although biologically derived double-stranded DNA can be used to realize DNA hydrogels with a high DNA content, similar mechanical properties can be achieved through the use of DNA nanostructures using lower quantities of DNA, effectively reducing the costs associated with the fabrication of DNA hydrogels. In this context, hybrid DNA hydrogels have been developed to address the cost issue while simultaneously utilizing the programmable nature of DNA for mechanically tunable biomaterials. In fact, hybrid hydrogels offer vast accessibility to diverse mechanical properties for DNA-based biomaterials. In contrast, pure DNA hydrogels lead to less stable mechanical characteristics. As a result, this somewhat limits their potential applications, despite their inherent thermal-responsiveness, thixotropic properties, and being advantageous for injectable therapeutics and bioprinting. Enhancing their stability under physiological conditions is clearly essential for utilizing DNA hydrogels in applications in vivo, as ensuring such stability will be crucial in preserving the designed mechanical properties for these applications. In view of in vivo drug release applications, the mechanical properties of DNA hydrogels are paramount for the hydrogel injection process, whereas in vitro applications can take advantage of DNA hydrogels for cell culturing in the absence of serum. Considerable efforts are being made to address the primary challenges encountered in implementing mechanically programmable DNA hydrogels, including cost and gel stability. Although design principles have achieved a considerable level of standardization in the fabrication of DNA origami thanks to the development of manual and automated design software, in the case of DNA hydrogels a similar standardized design is yet to be achieved. Thus, a current goal is compiling diverse designs for DNA hydrogels from a mechanical standpoint, which can be achieved by analyzing the spectrum of available DNA hydrogel designs and conducting a systematic examination of each design parameter programmed within a similar system in order to establish the desired essential design principles [72].

### 3.1. G-Quartet-Based Gels

**Figure 3:**
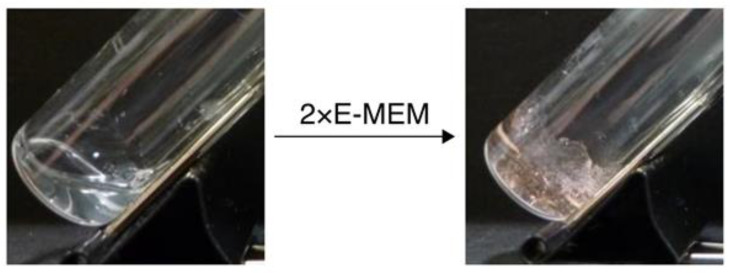
Photographs of 20 wt% L4.6k-dG4 before (**left**) and after (**right**) the addition of an equal volume of 2 × Eagle′s minimal essential medium (E-MEM). The pictures in Figure 3 were adapted from https://www.mdpi.com/2073-4360/11/10/1607 (accessed on 28 November 2023).

Guanosine, a biomolecule possessing hydrogen bond receptors and donors on its purine ring, exhibits self-assembly capabilities, forming chiral G-quartets [73] which serve as effective templates for developing supramolecular materials. Guanosine-containing compounds are also known to form gels in aqueous solution through reversible G-driven self-assembly [74,75]. Typically, acidic pH and low temperatures favor such gel formation. However, by combining binary mixtures of guanosine and 5′-guanosine monophosphate, it was possible to generate stable gels at neutral pH, and the temperature range for gelation was modulated using different proportions of hydrophobic guanosine and hydrophilic guanosine monophosphate within the gelator mixture. In this respect, the gelation behavior was investigated at 60 °C and within the temperature range of 5–40 °C, at a pH of 7.2, employing CD spectroscopy, CD thermal melting experiments, and visual detection [74]. The investigation revealed that solutions with higher amounts of hydrophilic gelator showed characteristics similar to solutions of pure guanosine monophosphate, while those with higher guanosine/guanosine monophosphate ratios formed stable gels across the entire temperature range. Notably, thermo-associative properties were displayed by solutions falling between these two extremes that underwent distinct transitions to a gel state only at elevated temperatures, while remaining liquid at refrigerator temperatures. Modulating the guanosine/guanosine monophosphate ratio and the overall concentration of guanosine compounds caused a shift in the onset of the gelation process towards higher temperatures, ranging between 20 and 40 °C [74]. These changes also led to a narrowed temperature range in which the gel phase was observed and enhanced the reversibility of the phase transitions. Combining reversible behavior, self-assembly, and adaptability within biologically relevant pH conditions and temperature ranges displays favorable potential for constructing cost-effective materials utilizable in various applications across nanotechnological, medical, biological, and analytical fields [74]. Chiral supramolecular gels found applications in circularly polarized luminescence [76,77,78,79], chiral recognition, and asymmetric catalysis. G-quartet-based ionogels [80] were fabricated without the involvement of metal ions by simply dissolving guanosine in an ionic liquid, ethylammonium nitrate, where the cations of the ionic liquid acted as stabilizers for the G-quartets. The addition of Sr^2+^ or K^+^ decreased the required guanosine concentration for the formation of G-quartet-based gels in the investigated ionic liquid. Sr^2+^ was able to disrupt the homopolar stacking of G-quartets, which determined the initiation of heteropolar stacking, thereby modifying the overall chirality of the G-quartet-based systems. Such ionogels exhibited circularly polarized luminescence without need of adding any supplementary luminescent materials, producing left-handed circularly polarized luminescence for K^+^ and right-handed in the case of Sr^2+^, respectively. Altering the metal ion proportions proved effective in modulating the chirality and other circularly polarized luminescence features of the hybrid G-quartet-based systems. The described ionogels, which can be easily fabricated, offer controllable chirality and circularly polarized luminescence characteristics, with promise for constructing devices able to emit polarized light [81]. Thus, G-quartet-based hydrogels (sometimes referred to as G-quadruplex hydrogels), can be defined as self-assembled supramolecular hydrogels realized starting from guanine derivatives. Such materials constitute a biomimetic hydrogel family endowed with diverse applications in the biomedical field, with utility in tissue engineering, drug delivery, and biosensing, thanks to their favorable properties of customizable multifunctionality, cost-effective large-scale manufacturing, biocompatibility, and biodegradability. The exploration into the biomedical applications of these hydrogels is paramount to facilitate their potential translation into industrial or clinical applications [82]. G-quartet-based gels are at the origin of the creation of intelligent biomaterials combining the outstanding biocompatibility of G-quartet-based systems and the unique biological and nanotechnological features of hydrogels, such as exceptional biodegradability, high water content, high water retention, hydrophilicity, and flexibility. This justifies why G-quartet-based hydrogels find extensive application across diverse research fields, with great attention being paid to their preparation techniques and their different possible applications. Notably, G-quartet-based hydrogels demonstrated their versatility in biomaterials, biomedicine, biosensing, and biocatalysis [83]. Apart from G-quartet systems based on a single guanine-containing moiety, entire oligonucleotides carrying multiple G bases able to form G-quadruplex [84,85,86,87,88,89,90] were also investigated in view of utilizing them in gel fabrication [91]. For example, Na^+^-responsive DNA quadruplex hydrogels, employing G-quadruplexes as binding points within a polyethylene glycol (PEG) network were realized and utilized as cell culture substrates [91]. From a chemical perspective, an oligodeoxynucleotide–PEG conjugate, indicated as L4.6k-dG4 (Figure 3), was synthesized and subsequently used to fabricate the above-mentioned hydrogel [91]. More in detail, the oligonucleotide tract containing four deoxyguanosine residues (hence the ‘dG4’ in its name) was prepared through a modified high-efficiency liquid-phase synthesis protocol for oligonucleotides. When the conjugate solution was added to an equal volume of cell culture medium, a stiff hydrogel was obtained (Figure 3), which was labelled G-quadruplex hydrogel. The presence of Na^+^ led the tetradeoxyguanosine segments to form G-quadruplex structures, which also determined a solidification of the solution into a stable hydrogel which was found suitable for cell culture [91]. 

Notably, the conjugate itself did not exhibit any cytotoxicity, and the resultant hydrogel maintained appreciable stability under the explored cell culture conditions. Notably, when culturing L929 fibroblast cells within the G-quadruplex hydrogel, the fibroblasts remained spherical in shape and inactive for a week, without any proliferation, although still viable. Over time, they gradually settled through the gel, potentially due to the reversible nature of G-quadruplex’s formation, leading to the slow reorganization of the gelators. Once the fibroblast cells reached the underlying glass surface, they initiated their spreading and growing activities [91].

### 3.2. RNA-Based Gels

Similar to DNA, certain RNAs demonstrated promise for forming gels through self-assembly mechanisms [92]. In particular, RNA hydrogels are created by the structural properties and molecular interacting propensity of specific RNA sequences that allow them to assemble into three-dimensional structures. A typical RNA sequence able to form hydrogels typically involves short ribonucleic tracts with complementary nucleobase sequences that can interact, forming stable structures. These RNA sequences may contain motifs that enable them to self-assemble under suitable conditions of pH, temperature, and ionic strength. Hydrogel-forming RNA sequences often possess unique structural elements, including self-complementary regions or specific stem-loop structures. These sequences allow the formation of double-stranded regions, which ultimately provokes the organization of a gel-like network. Despite the chemical instability and the technological limitations related to RNA usage, the versatile functionalities and the abundant structural elements of ribonucleic acids have led to RNA utilization in biomedical and nanotechnological strategies. For instance, a stable RNA hydrogel was engineered through a sequential process that involved both the amplification method known as rolling circle transcription and ligation. This process led to the formation of RNA G-quadruplexes with catalytic capabilities and able to enhance the expression of various proteins within separated translation environments formed by distinct compartments. These findings of the mentioned study suggest that RNA hydrogels can be useful tools for cell-free protein expression and other potential applications that broaden the scope of RNA-related research, disclosing new scenarios for practical applications of these nucleic acids [93]. Importantly, creating an RNA-based hydrogel presents challenges due to the inherent instability of RNA itself, unlike DNA hydrogels, whose nucleic constituents are famously more stable than their ribonucleic counterparts. Aimed at overcoming these drawbacks, a hybrid RNA–DNA hydrogel was realized using a stepwise dual enzymatic polymerization strategy. More in detail, by combining functional DNA aptamers with short hairpin RNAs, it was possible to obtain the hybrid RNA–DNA hydrogel with the nucleic constituents being crucial for targeting specific elements and simultaneously influencing the mechanical properties of the resultant hydrogel. The hybrid hydrogel displayed remarkable traits such as high injectability, durability, and softness and, under physiological conditions, emulated microtubule structures. Moreover, the hybrid RNA–DNA hydrogel was engineered to sequentially release functional cargos of small interfering RNA–aptamer complexes. Additionally, specific sites responsive to restriction enzymes were integrated within the hybrid hydrogel structure to enhance the release of the above complex. This innovative approach offered an efficient platform for controlled RNA delivery, ensuring a double-controlled release: firstly, the release of a small interfering RNA–aptamer complex from the hydrogel and, subsequently, the release of a small interfering RNA from the complex. Remarkably, this dual-controlled RNA release system demonstrated exceptional potential in advanced RNA therapeutic approaches [94]. Apart from single-stranded and quadruplex RNA, RNA triple helix is also a structure endowed with considerable importance in biomedicine [95,96,97]. Moreover, in a past study, RNA-triple-helix structures served for the fabrication of self-assembled hydrogel scaffolds designed for modulating the levels of microRNAs within the tumor microenvironment [98]. Famously, the efficacy of microRNAs in cancer therapy is often hindered by the poor efficiency of the used delivery systems. On the other hand, in the same study, self-assembled RNA structures, consisting of two different microRNAs, an inhibitor of an onco-microRNA and a tumor suppressor microRNA mimic, exhibited significant promise for effectively combating tumors by exerting a synergistic anti-cancer action [98]. By linking RNA triple helices to dendrimers, it was possible to obtain stable triplex nanoparticles, which, upon treatment with dextran aldehyde, originated an RNA-triple-helix adhesive scaffold able to chemically adhere to natural tissue amines within the tumor. Notably, the self-assembled RNA-triple-helix conjugates retained their functionality both in vitro and in vivo [98]. The implantation of these conjugates in a breast cancer mouse model resulted in an approximately 90% reduction in tumor size two weeks post-hydrogel implantation. Overall, the above findings collectively suggest that RNA-triple-helix hydrogels could serve as an efficacious anti-cancer platform by locally regulating the expression of endogenous microRNAs in cancerous tissues [98]. The systemic administration of anti-cancer drugs is a conventional approach used in cancer combination therapy that leads to physiological toxicity and potential harm to healthy cells due to the lack of intelligent drug targeting as well as the toxicity of the drug carriers. A triple-combination nanosystem, employing multifunctional RNA hydrogels of nanometric size was developed and used in gene delivery therapy, chemotherapy, and phototherapy [99]. By utilizing DNA nanotechnology and the above-mentioned rolling circle transcription amplification method, three lung cancer inhibitory microRNAs (let-7a, 34a, and 145) were integrated into a single RNA hydrogel nanoparticle, resulting in the simultaneous silencing of three specific targeted mRNAs [99]. Additionally, the RNA hydrogel was able to carry doxorubicin, employed as a chemotherapeutic drug model, along with TMPyP4, a photosensitizer, and deliver it to cancer cells. These molecular cargoes exhibited a synergistic anti-cancer effect, as demonstrated in experiments using multidrug-resistant cancer cells. By making use of an aptamer sequence S6, modified with cholesterol, the RNA nanohydrogels were condensed to an appropriate size without any need for polyelectrolyte condensation agents and demonstrated specific targeting towards cancer cells [99]. Therefore, significant quantities of microRNA, along with the photosensitizer and the chemotherapeutic drug, were specifically delivered to cancer cells, showing synergistic therapeutic activity both in vitro and in vivo. As a result, the extraordinary potential of the triple-combination therapy nanosystems based on RNA gels for overcoming multidrug resistance associated with chemotherapy-related gene malfunctions was clearly demonstrated, and this approach offers promising prospects for synergistic and multifunctional cancer therapeutic strategies [99]. Hypoxia is a well-known factor capable of driving tumor metastasis and contribute to the spread of cancer cells from their original site, often leading to cancer reemergence. A self-assembled RNA hydrogel was found capable of effectively delivering a combination of synergistic agents—short hairpin RNA, DNA CpG, doxorubicin, and MnO_2_-loaded photodynamic agent ‘chlorin e6 (Ce6)’ (abbreviated as MnO_2_@Ce6)—into MDA-MB-231 breast cancer cells. This RNA hydrogel comprised both an anti-metastatic microRNA (microRNA-182) and a tumor suppressor microRNA (microRNA-205), demonstrating a remarkable synergistic effect in tumor eradication. As for its design, the hydrogel could be disassembled by the Dicer enzyme, which facilitated the release of the loaded therapeutic agents while concurrently catalyzing the decomposition of endogenous tumor H_2_O_2_, which is useful for alleviating tumor hypoxia. Consequently, a remarkable synergistic therapeutic effect was observed combining chemo-photodynamic therapeutics in the above approach, which triggered a series of favorable anti-tumor immune responses. Furthermore, the RNA hydrogel drug delivery system, suitably modified with aptamers to target breast cancer cells, demonstrated advantageous properties of biocompatibility while showing low cytotoxicity. This approach could be extended to design other microRNA carriers, combined with various therapeutic combinations, to target human cancer, thus potentially overcoming the limitations of current cancer therapies [100].

### 3.3. Comparing DNA and RNA-Based Systems

Owing to their composition and structure, DNA hydrogels are formed through the self-assembly of DNA into three-dimensional frameworks, with the assembly being driven by molecular interactions such as base stacking and complementary base pairing. The ability to form hydrogels is often observed for short DNA sequences bearing complementary base sequences at their ends. Hydrogel formation involves the hybridization of these complementary sequences, resulting in double-stranded DNA structures and the subsequent assembly of three-dimensional DNA networks. RNA hydrogels, like DNA hydrogels, are created through base-driven self-assembly mechanisms and are typically formed by short ribonucleic tracts with complementary nucleobase sequences, allowing the formation of stable structures. Additionally, RNA sequences may contain specific motifs enabling self-assembly under suitable conditions of pH, temperature, and ionic strength. These structural elements may include self-complementary regions or specific stem-loop structures. Another property needing to be evaluated with great attention is the stability of nucleic acid gels and of their oligonucleotide components. DNA is endowed with stability properties sufficient for different biomedical and technological applications. Moreover, DNA hydrogels benefit from the unique mechanical, biochemical, and biocompatibility characteristics of DNA materials. They offer controlled composition and mechanical properties, making them suitable for various biomedical applications. On the other hand, RNA, in general, is less stable than DNA, and, thus, a major challenge in realizing RNA hydrogels lies in overcoming the inherent instability of ribonucleic acid. Strategies such as hybrid RNA–DNA hydrogels have been employed to combine the functionalities of both nucleic acids, providing a more stable platform. As for their applications, DNA hydrogels have uses in tissue engineering, drug delivery, and biosensing, among others. They are programmable and responsive to external stimuli, which expands their potential applications to include shape-memory materials and detection devices. RNA hydrogels have been engineered for controlled RNA delivery in gene delivery therapy, phototherapy, and chemotherapy. Overall, RNA hydrogels have demonstrated particular promise for cancer therapy, providing a platform for synergistic and multifunctional approaches. The design principles for DNA hydrogels have achieved a considerable level of standardization. However, challenges such as the steric availability of crosslinking points, structural rigidity, and melting temperature need to be considered for specific properties. Designing RNA hydrogels presents challenges due to the inherent instability of RNA, and strategies like the use of hybrid or multi-component hydrogels are currently under investigation as ways to overcome these design challenges. In summary, both systems can form hydrogels through self-assembly, but DNA hydrogels are generally more stable than their RNA-based counterparts. Both have unique properties of self-assembly that make them valuable in various applications, and ongoing research is addressing design challenges to enhance their utility.

## 4. Conclusions

The exploration of the self-assembly of nucleobase-bearing amino acid systems, such as nucleoamino acids and nucleopeptides, as well as the investigation of nucleic acid-based gels, including DNA hydrogels, G-quartet-based gels, and RNA-based gels, uncovers a wide landscape of nanosystems and biomaterials with extensive potential in nanotechnology and biomedicine. As for the family of nucleopeptides, the fusion of amino acids with DNA or RNA bases creates a bridge between peptides and nucleobases, which provides unique biomolecular binding capabilities as well as advantageous self-assembly properties employable in gel construction, producing nucleoamino acids, whether synthetic or natural, and nucleopeptides molecules with great potential in medicine, nanotechnology, and materials science. Concurrently, the investigation into nucleic acid-based gels, enriched by functional nucleic acids, reveals promising avenues for innovative biomedical applications. Remarkably, functional nucleic acids have further enabled the creation of hydrogels based on aptamers, i-motif nanostructures, DNAzymes, CpG oligodeoxynucleotides, and siRNAs, enriching these materials with enhanced catalytic capabilities, molecular recognition, and bioanalytical and therapeutic potential [101]. Summarizing the key findings emerged from the papers reviewed in the present work, hydrogels formed by nucleopeptides with self-assembling capabilities offer substantial advantages, including biocompatibility, ease of synthesis, and modulable properties. These hydrogels show promise in drug delivery, tissue engineering, and various biomedical applications. Also, DNA hydrogels possess unique properties, such as biocompatibility and responsiveness to stimuli, making them valuable in tissue engineering, biosensing, and drug delivery. Ongoing challenges, including the need for standardized design principles and stability improvement under physiological conditions, indicate the necessity for further research on DNA gels. The ability to design DNA hydrogels with specific mechanical and responsive properties is envisaged to open new avenues for tailored gel-based biomaterials. On the other hand, G-quartet-based hydrogels demonstrate versatility in various applications, including circularly polarized luminescence and chiral recognition. The modulation of gelation behavior through hydrophobic/hydrophilic ratios offers control over temperature-dependent gelation, enhancing the adaptability of the materials. Owing to the consequent implications of these molecular systems, G-quartet-based hydrogels show potential in tissue engineering, drug delivery, and biosensing and are endowed with good biocompatibility and multifunctionality. RNA hydrogels, despite facing challenges due to instability, present opportunities in controlled RNA delivery and cancer therapy. Strategies using hybrid RNA–DNA hydrogels and RNA triple helices seem to address stability concerns and demonstrate great promise for advanced therapeutic approaches. Various combination approaches applied to RNA gels show potential in overcoming multidrug resistance in cancer therapy. Future research should be devoted to understanding intricate interactions governing nucleobase-driven self-assembly. Exploring a broader repertoire of nucleobase types and sequences and their applications in specific medical and materials-based contexts will enhance therapeutic and technological outcomes. In summary, the take-home message of this review is that the intersection of peptide- and nucleic acid-based gels, enriched by guanine-based assemblies, offers a vast spectrum of gels that are intelligent biomaterials with potential applications ranging from drug delivery to tissue engineering and more. Despite several challenges, including the urgent need for standardized design principles and for the stability enhancement of natural nucleic acids, the ongoing research holds exciting possibilities for the development of advanced nucleobase-containing gel-based biomaterials.

## Data Availability

No data available.

## References

[1] Djabourov M. (1991). Gelation—A review. Polym. Int..

[2] Rogovina L.Z., Vasil’ev V.G., Braudo E. (2008). Definition of the concept of polymer gel. Polym. Sci. Ser. C.

[3] Yamauchi A. (2001). Gels: Introduction. Gels Handbook.

[4] Draper E.R., Adams D.J. (2017). Low-molecular-weight gels: The state of the art. Chem.

[5] Banerjee S., Bhattacharya S. (2012). Food gels: Gelling process and new applications. Crit. Rev. Food Sci. Nutr..

[6] Siddiqui S.A., Alvi T., Biswas A., Shityakov S., Gusinskaia T., Lavrentev F., Dutta K., Khan M.K.I., Stephen J., Radhakrishnan M. (2022). Food gels: Principles, interaction mechanisms and its microstructure. Crit. Rev. Food Sci. Nutr..

[7] Nazir A., Asghar A., Maan A.A. (2017). Food gels: Gelling process and new applications. Advances in Food Rheology and Its Applications.

[8] Nayak A.K., Das B. (2018). Introduction to polymeric gels. Polymeric Gels.

[9] Vashist A., Vashist A., Gupta Y., Ahmad S. (2014). Recent advances in hydrogel based drug delivery systems for the human body. J. Mater. Chem. B.

[10] Chamkouri H., Chamkouri M. (2021). A review of hydrogels, their properties and applications in medicine. Am. J. Biomed. Sci. Res..

[11] Hwang H.S., Lee C.-S. (2023). Recent progress in hyaluronic-acid-based hydrogels for bone tissue engineering. Gels.

[12] Kopeček J. (2007). Hydrogel biomaterials: A smart future?. Biomaterials.

[13] Shakeel S., Karim S., Ali A. (2006). Peptide nucleic acid (PNA)—A review. J. Chem. Technol. Biotechnol. Int. Res. Process Environ. Clean Technol..

[14] Rodrigues T., Curti F., Leroux Y.R., Barras A., Pagneux Q., Happy H., Kleber C., Boukherroub R., Hasler R., Volpi S. (2023). Discovery of a Peptide Nucleic Acid (PNA) aptamer for cardiac troponin I: Substituting DNA with neutral PNA maintains picomolar affinity and improves performances for electronic sensing with graphene field-effect transistors (gFET). Nano Today.

[15] Pradeep S.P., Malik S., Slack F.J., Bahal R. (2023). Unlocking the potential of chemically modified peptide nucleic acids for RNA-based therapeutics. RNA.

[16] Chu T.-W., Feng J., Yang J., Kopeček J. (2015). Hybrid polymeric hydrogels via peptide nucleic acid (PNA)/DNA complexation. J. Control. Release.

[17] Park S.J., Park S.M., Kim W.-k., Lee J. (2023). Hydrogel-based thermosensor using peptide nucleic acid and PEGylated graphene oxide. Anal. Chim. Acta.

[18] Langford G.J., Raeburn J., Ferrier D.C., Hands P.J., Shaver M.P. (2018). Morpholino oligonucleotide cross-linked hydrogels as portable optical oligonucleotide biosensors. ACS Sens..

[19] Agrawal N.K., Allen P., Song Y.H., Wachs R.A., Du Y., Ellington A.D., Schmidt C.E. (2020). Oligonucleotide-functionalized hydrogels for sustained release of small molecule (aptamer) therapeutics. Acta Biomater..

[20] Liu J. (2011). Oligonucleotide-functionalized hydrogels as stimuli responsive materials and biosensors. Soft Matter.

[21] Bhattacharyya T., Saha P., Dash J. (2018). Guanosine-derived supramolecular hydrogels: Recent developments and future opportunities. ACS Omega.

[22] Ye X., Li X., Shen Y., Chang G., Yang J., Gu Z. (2017). Self-healing pH-sensitive cytosine-and guanosine-modified hyaluronic acid hydrogels via hydrogen bonding. Polymer.

[23] Merino-Gómez M., Godoy-Gallardo M., Wendner M., Mateos-Timoneda M.A., Gil F.J., Perez R.A. (2023). Optimization of guanosine-based hydrogels with boric acid derivatives for enhanced long-term stability and cell survival. Front. Bioeng. Biotechnol..

[24] Godoy-Gallardo M., Merino-Gómez M., Mateos-Timoneda M.A., Eckhard U., Gil F.J., Perez R.A. (2023). Advanced Binary Guanosine and Guanosine 5′-Monophosphate Cell-Laden Hydrogels for Soft Tissue Reconstruction by 3D Bioprinting. ACS Appl. Mater. Interfaces.

[25] Tripathi M., Sharma R., Hussain A., Kumar I., Sharma A.K., Sarkar A. (2023). Hydrogels and their combination with lipids and nucleotides. Sustainable Hydrogels.

[26] Peters G.M., Davis J.T. (2016). Supramolecular gels made from nucleobase, nucleoside and nucleotide analogs. Chem. Soc. Rev..

[27] Godeau G., Brun C., Arnion H., Staedel C., Barthélémy P. (2010). Glycosyl-nucleoside fluorinated amphiphiles as components of nanostructured hydrogels. Tetrahedron Lett..

[28] Godoy-Gallardo M., Merino-Gómez M., Matiz L.C., Mateos-Timoneda M.A., Gil F.J., Perez R.A. (2022). Nucleoside-based supramolecular hydrogels: From synthesis and structural properties to biomedical and tissue engineering applications. ACS Biomater. Sci. Eng..

[29] Ignatowska J., Mironiuk-Puchalska E., Grześkowiak P., Wińska P., Wielechowska M., Bretner M., Karatsai O., Rędowicz M.J., Koszytkowska-Stawińska M. (2020). New insight into nucleo α-amino acids–Synthesis and SAR studies on cytotoxic activity of β-pyrimidine alanines. Bioorg. Chem..

[30] More J.C., Troop H.M., Dolman N.P., Jane D.E. (2003). Structural requirements for novel willardiine derivatives acting as AMPA and kainate receptor antagonists. Br. J. Pharmacol..

[31] Mik V., Mičková Z., Doležal K., Frébort I., Pospisil T. (2017). Activity of (+)-Discadenine as a plant cytokinin. J. Nat. Prod..

[32] Xu Q., Song B., Liu F., Song Y., Chen P., Liu S., Krishnan H.B. (2018). Identification and characterization of β-Lathyrin, an abundant glycoprotein of grass pea (*Lathyrus sativus* L.), as a potential allergen. J. Agric. Food Chem..

[33] Roviello G.N., Gaetano S.D., Capasso D., Cesarani A., Bucci E.M., Pedone C. (2010). Synthesis, spectroscopic studies and biological activity of a novel nucleopeptide with Moloney murine leukemia virus reverse transcriptase inhibitory activity. Amino Acids.

[34] Roviello G.N., Musumeci D., De Cristofaro A., Capasso D., Di Gaetano S., Bucci E.M., Pedone C. (2009). Alternate dab-aeg PNAs: Synthesis, nucleic acid binding studies and biological activity. Mol. Biosyst..

[35] Roviello G., Musumeci D., Castiglione M., Bucci E., Pedone C., Benedetti E. (2009). Solid phase synthesis and RNA-binding studies of a serum-resistant nucleo-ε-peptide. J. Pept. Sci. Off. Publ. Eur. Pept. Soc..

[36] Roviello G.N., Moccia M., Sapio R., Valente M., Bucci E., Castiglione M., Pedone C., Perretta G., Benedetti E., Musumeci D. (2006). Synthesis, characterization and hybridization studies of new nucleo-γ-peptides based on diaminobutyric acid. J. Pept. Sci. Off. Publ. Eur. Pept. Soc..

[37] Roviello V., Musumeci D., Mokhir A., Roviello G.N. (2021). Evidence of protein binding by a nucleopeptide based on a thyminedecorated L-diaminopropanoic acid through CD and in silico studies. Curr. Med. Chem..

[38] Hoschtettler P., Pickaert G., Carvalho A., Averlant-Petit M.-C., Stefan L. (2023). Highly Synergistic Properties of Multicomponent Hydrogels Thanks to Cooperative Nucleopeptide Assemblies. Chem. Mater..

[39] Musumeci D., Ullah S., Ikram A., Roviello G.N. (2022). Novel insights on nucleopeptide binding: A spectroscopic and In Silico investigation on the interaction of a thymine-bearing tetrapeptide with a homoadenine DNA. J. Mol. Liq..

[40] Boback K., Bacchi K., O’Neill S., Brown S., Dorsainvil J., Smith-Carpenter J.E. (2020). Impact of C-terminal chemistry on self-assembled morphology of guanosine containing nucleopeptides. Molecules.

[41] Datta A. (2013). Synthetic Studies on Antifungal Peptidyl Nucleoside Antibiotics. Chem. Synth. Nucleoside Analog..

[42] Swinehart W., Deutsch C., Sarachan K.L., Luthra A., Bacusmo J.M., de Crécy-Lagard V., Swairjo M.A., Agris P.F., Iwata-Reuyl D. (2020). Specificity in the biosynthesis of the universal tRNA nucleoside N6-threonylcarbamoyl adenosine (t6A)—TsaD is the gatekeeper. RNA.

[43] Roviello G.N., Benedetti E., Pedone C., Bucci E.M. (2010). Nucleobase-containing peptides: An overview of their characteristic features and applications. Amino Acids.

[44] Kurbasic M., Garcia A.M., Viada S., Marchesan S. (2021). Tripeptide self-assembly into bioactive hydrogels: Effects of terminus modification on biocatalysis. Molecules.

[45] Snip E., Koumoto K., Shinkai S. (2002). Gel formation properties of a uracil-appended cholesterol gelator and cooperative effects of the complementary nucleobases. Tetrahedron.

[46] Marchesan S., Vargiu A.V., Styan K.E. (2015). The Phe-Phe motif for peptide self-assembly in nanomedicine. Molecules.

[47] Dinesh B., Squillaci M.A., Ménard-Moyon C., Samorì P., Bianco A. (2015). Self-assembly of diphenylalanine backbone homologues and their combination with functionalized carbon nanotubes. Nanoscale.

[48] Roviello G.N. (2018). Novel insights into nucleoamino acids: Biomolecular recognition and aggregation studies of a thymine-conjugated l-phenyl alanine. Amino Acids.

[49] Scognamiglio P.L., Riccardi C., Palumbo R., Gale T.F., Musumeci D., Roviello G.N. (2023). Self-assembly of thyminyl l-tryptophanamide (TrpT) building blocks for the potential development of drug delivery nanosystems. J. Nanostructure Chem..

[50] Guida S., Arginelli F., Farnetani F., Ciardo S., Bertoni L., Manfredini M., Zerbinati N., Longo C., Pellacani G. (2021). Clinical applications of in vivo and ex vivo confocal microscopy. Appl. Sci..

[51] Li X., Kuang Y., Lin H.-C., Gao Y., Shi J., Xu B. (2011). Supramolecular nanofibers and hydrogels of nucleopeptides. Angew. Chem. Int. Ed. Engl..

[52] Yuan D., Du X., Shi J., Zhou N., Zhou J., Xu B. (2015). Mixing biomimetic heterodimers of nucleopeptides to generate biocompatible and biostable supramolecular hydrogels. Angew. Chem..

[53] Ewert E., Pospieszna-Markiewicz I., Szymańska M., Kurkiewicz A., Belter A., Kubicki M., Patroniak V., Fik-Jaskółka M.A., Roviello G.N. (2023). New N4-Donor Ligands as Supramolecular Guests for DNA and RNA: Synthesis, Structural Characterization, In Silico, Spectrophotometric and Antimicrobial Studies. Molecules.

[54] Baek K., Noblett A.D., Ren P., Suggs L.J. (2020). Self-assembled nucleo-tripeptide hydrogels provide local and sustained doxorubicin release. Biomater. Sci..

[55] Giraud T., Bouguet-Bonnet S., Marchal P., Pickaert G., Averlant-Petit M.-C., Stefan L. (2020). Improving and fine-tuning the properties of peptide-based hydrogels via incorporation of peptide nucleic acids. Nanoscale.

[56] Palumbo R., Simonyan H., Roviello G.N. (2023). Advances in Amino Acid-Based Chemistry. Pharmaceuticals.

[57] Du X., Zhou J., Li X., Xu B. (2017). Self-assembly of nucleopeptides to interact with DNAs. Interface Focus.

[58] Roviello G.N., Musumeci D., Bucci E.M., Pedone C. (2011). Evidences for supramolecular organization of nucleopeptides: Synthesis, spectroscopic and biological studies of a novel dithymine L-serine tetrapeptide. Mol. BioSyst..

[59] Roviello G.N., Ricci A., Bucci E.M., Pedone C. (2011). Synthesis, biological evaluation and supramolecular assembly of novel analogues of peptidyl nucleosides. Mol. BioSyst..

[60] Giraud T., Hoschtettler P., Pickaert G., Averlant-Petit M.-C., Stefan L. (2022). Emerging low-molecular weight nucleopeptide-based hydrogels: State of the art, applications, challenges and perspectives. Nanoscale.

[61] Scognamiglio P.L., Platella C., Napolitano E., Musumeci D., Roviello G.N. (2021). From prebiotic chemistry to supramolecular biomedical materials: Exploring the properties of self-assembling nucleobase-containing peptides. Molecules.

[62] Wang H., Feng Z., Xu B. (2019). Supramolecular assemblies of peptides or nucleopeptides for gene delivery. Theranostics.

[63] Wang H., Feng Z., Qin Y., Wang J., Xu B. (2018). Nucleopeptide assemblies selectively sequester ATP in cancer cells to increase the efficacy of doxorubicin. Angew. Chem..

[64] Ghosh S., Ghosh T., Bhowmik S., Patidar M.K., Das A.K. (2023). Nucleopeptide-coupled injectable bioconjugated guanosine-quadruplex hydrogel with inherent antibacterial activity. ACS Appl. Bio Mater..

[65] Noblett A.D., Baek K., Suggs L.J. (2021). Controlling Nucleopeptide Hydrogel Self-Assembly and Formation for Cell-Culture Scaffold Applications. ACS Biomater. Sci. Eng..

[66] Baek K., Noblett A.D., Ren P., Suggs L.J. (2019). Design and characterization of nucleopeptides for hydrogel self-assembly. ACS Appl. Bio Mater..

[67] Zhang Z., Han J., Pei Y., Fan R., Du J. (2018). Chaperone copolymer-assisted aptamer-patterned DNA hydrogels for triggering spatiotemporal release of protein. ACS Appl. Bio Mater..

[68] Morán M.C., Infante M.R., Miguel M.G., Lindman B., Pons R. (2010). Novel biocompatible DNA gel particles. Langmuir.

[69] Shi J., Shi Z., Dong Y., Wu F., Liu D. (2020). Responsive DNA-based supramolecular hydrogels. ACS Appl. Bio Mater..

[70] Wei Y., Wang K., Luo S., Li F., Zuo X., Fan C., Li Q. (2022). Programmable DNA hydrogels as Artificial extracellular matrix. Small.

[71] Ma G., Zhang K., Wang H., Liang Z., Zhou L., Yan B. (2021). Versatile synthesis of a highly porous DNA/CNT hydrogel for the adsorption of the carcinogen PAH. Chem. Commun..

[72] Bush J., Hu C.-H., Veneziano R. (2021). Mechanical properties of DNA hydrogels: Towards highly programmable biomaterials. Appl. Sci..

[73] Gao C., Zhang Z., Zhang X., Chen J., Chen Y., Zhao C., Zhao L., Feng L. (2022). A molecular crowding thermo-switchable chiral G-quartet hydrogel with circularly polarized luminescence property. Soft Matter.

[74] Yu Y., Nakamura D., DeBoyace K., Neisius A.W., McGown L.B. (2008). Tunable thermoassociation of binary guanosine gels. J. Phys. Chem. B.

[75] Davis J.T., Spada G.P. (2007). Supramolecular architectures generated by self-assembly of guanosine derivatives. Chem. Soc. Rev..

[76] Longhi G., Castiglioni E., Koshoubu J., Mazzeo G., Abbate S. (2016). Circularly polarized luminescence: A review of experimental and theoretical aspects. Chirality.

[77] Imai Y. (2020). Generation of Circularly Polarized Luminescence by Symmetry Breaking. Symmetry.

[78] Yang G., Zhang S., Hu J., Fujiki M., Zou G. (2019). The chirality induction and modulation of polymers by circularly polarized light. Symmetry.

[79] Zou C., Qu D., Jiang H., Lu D., Ma X., Zhao Z., Xu Y. (2019). Bacterial cellulose: A versatile chiral host for circularly polarized luminescence. Molecules.

[80] Le Bideau J., Viau L., Vioux A. (2011). Ionogels, ionic liquid based hybrid materials. Chem. Soc. Rev..

[81] Qi P., Li X., Huang Z., Liu Y., Song A., Hao J. (2021). G-quadruplex-based ionogels with controllable chirality for circularly polarized luminescence. Colloids Surf. A Physicochem. Eng. Asp..

[82] Li Y., Chi J., Xu P., Dong X., Le A.-T., Shi K., Liu Y., Xiao J. (2023). Supramolecular G-quadruplex hydrogels: Bridging fabrication to biomedical application. J. Mater. Sci. Technol..

[83] Fang J., Zheng L., Liu Y., Peng Y., Yang Q., Huang Y., Zhang J., Luo L., Shen D., Tan Y. (2023). Smart G-quadruplex hydrogels: From preparations to comprehensive applications. Int. J. Biol. Macromol..

[84] Roxo C., Kotkowiak W., Pasternak A. (2019). G-quadruplex-forming aptamers—Characteristics, applications, and perspectives. Molecules.

[85] Bidzinska J., Cimino-Reale G., Zaffaroni N., Folini M. (2013). G-quadruplex structures in the human genome as novel therapeutic targets. Molecules.

[86] Asamitsu S., Obata S., Yu Z., Bando T., Sugiyama H. (2019). Recent progress of targeted G-quadruplex-preferred ligands toward cancer therapy. Molecules.

[87] Alessandrini I., Recagni M., Zaffaroni N., Folini M. (2021). On the road to fight cancer: The potential of G-quadruplex ligands as novel therapeutic agents. Int. J. Mol. Sci..

[88] Santos T., Salgado G.F., Cabrita E.J., Cruz C. (2021). G-quadruplexes and their ligands: Biophysical methods to unravel G-quadruplex/ligand interactions. Pharmaceuticals.

[89] Ruggiero E., Zanin I., Terreri M., Richter S.N. (2021). G-quadruplex targeting in the fight against viruses: An update. Int. J. Mol. Sci..

[90] Marzano M., Falanga A.P., Marasco D., Borbone N., D’Errico S., Piccialli G., Roviello G.N., Oliviero G. (2020). Evaluation of an analogue of the marine ε-PLL peptide as a ligand of G-quadruplex DNA structures. Mar. Drugs.

[91] Tanaka S., Yukami S., Hachiro Y., Ohya Y., Kuzuya A. (2019). Application of DNA quadruplex hydrogels prepared from polyethylene glycol-oligodeoxynucleotide conjugates to cell culture media. Polymers.

[92] Huang Z., Kangovi G.N., Wen W., Lee S., Niu L. (2017). An RNA aptamer capable of forming a hydrogel by self-assembly. Biomacromolecules.

[93] Ahn S.Y., Kim J., Vellampatti S., Oh S., Lim Y.T., Park S.H., Luo D., Chung J., Um S.H. (2022). Protein-Encoding Free-Standing RNA Hydrogel for Sub-Compartmentalized Translation. Adv. Mater..

[94] Han S., Park Y., Kim H., Nam H., Ko O., Lee J.B. (2020). Double controlled release of therapeutic RNA modules through injectable DNA–RNA hybrid hydrogel. ACS Appl. Mater. Interfaces.

[95] Brown J.A. (2020). Unraveling the structure and biological functions of RNA triple helices. Wiley Interdiscip. Rev. RNA.

[96] Conrad N.K. (2014). The emerging role of triple helices in RNA biology. Wiley Interdiscip. Rev. RNA.

[97] Maldonado R., Längst G. (2023). The chromatin–triple helix connection. Biol. Chem..

[98] Conde J., Oliva N., Atilano M., Song H.S., Artzi N. (2016). Self-assembled RNA-triple-helix hydrogel scaffold for microRNA modulation in the tumour microenvironment. Nat. Mater..

[99] Li J., Yuan D., Zheng X., Zhang X., Li X., Zhang S. (2020). A triple-combination nanotechnology platform based on multifunctional RNA hydrogel for lung cancer therapy. Sci. China Chem..

[100] Wang W., Liu X., Ding L., Jin H.J., Li X. (2021). Rna hydrogel combined with MnO2 nanoparticles as a nano-vaccine to treat triple negative breast cancer. Front. Chem..

[101] Ma Y., Duan X., Huang J. (2023). DNA Hydrogels as Functional Materials and Their Biomedical Applications. Adv. Funct. Mater..

